# Application of a Food-Based Nutritional Profiling System to Assess Diet Quality in Diet-Level Data: Evidence on Construct and Convergent Validity from the Hatoyama Cohort Study and Kusatsu Cohort Study

**DOI:** 10.1016/j.cdnut.2025.107630

**Published:** 2026-01-06

**Authors:** Tao Yu, Ryota Wakayama, Yuri Yokoyama, Hiroshi Murayama

**Affiliations:** 1 Research and Development Division, Meiji Co., Ltd., Tokyo, Japan; 2 Research Team for Social Participation and Healthy Aging, Tokyo Metropolitan Institute for Geriatrics and Gerontology, Tokyo, Japan

**Keywords:** nutrient profiling, diet quality, validation, older adults, epidemiology

## Abstract

**Background:**

Nutritional Profiling Systems (NPSs) are designed to classify foods by nutritional quality, but most validations occur at the food level. Their applicability to diet-level data from dietary questionnaires—commonly used in epidemiology—remains unclear.

**Objectives:**

The study aims to evaluate the construct and convergent validity of the Meiji NPS for Older Adults (MNPS-OA), the first NPS developed for older populations, when applied to diet-level data.

**Methods:**

Cross-sectional data from the Hatoyama Cohort Study and the Kusatsu Cohort Study involving 1102 Japanese adults aged ≥65 y were utilized. Dietary data were analyzed using a validated Brief Dietary History Questionnaire. Four MNPS-OA specifications were tested: *1*) original, *2*) without energy limits (WEL), *3*) without nutrient caps (WC), and *4*) without energy limit and nutrient caps (WEL-WC). Construct validity was assessed by intermodel correlations; convergent validity was evaluated against Healthy Eating Index-2015 (HEI-2015) and Nutrient-Rich Food Index 9.3 (NRF9.3).

**Results:**

Median MNPS-OA scores ranged from 124.5 (original) to 391.6 (WEL-WC). Correlations with HEI-2015 improved from *r* = 0.27 (original) to 0.58 (WEL-WC), and with NRF9.3 from *r* = 0.26 to 0.61. Removing nutrient caps substantially enhanced convergence, whereas removing energy limits had minimal effect.

**Conclusions:**

MNPS-OA can be adapted for diet-level assessment with targeted modifications. Eliminating nutrient caps markedly improves alignment with established diet quality indices, supporting its potential use in large-scale epidemiological studies and public health applications.

## Introduction

The WHO defines a nutrient profiling as “the science of classifying or ranking foods according to their nutritional composition for reasons related to preventing disease and promoting health” [1]. A Nutritional Profiling System (NPS) is a framework that assigns positive scores to nutrients and food components to encourage, and negative scores to those to limit, thereby quantifying the nutritional quality of foods [1,2].

Traditionally, NPSs have been developed on a per-food basis, using reference amounts such as 100 g, 100 mL, or 100 kcal [[3], [4], [5]] for general adult population. Their validity is often assessed by aggregating food-level scores into a diet-level score and comparing it with external indices or health outcomes [[6], [7], [8], [9], [10], [11]]. However, very few NPSs have been specifically developed for older adults—an age group with distinct health needs, unique nutritional intake patterns, and growing public health importance worldwide. Foods ranked by NPSs designed for adults may not adequately address the health needs of older adults; therefore, NPSs tailored to the specific health requirements of this age group are necessary. The Meiji NPS for Older Adults (MNPS-OA) was developed to address these challenges, drawing on existing NPS frameworks while aligning with Japan’s demographic structure, dietary habits among older adults, and nutritional intake patterns [2,12]. The algorithm design and numerical targets of MNPS-OA have been published in peer-reviewed papers and are expected to be applied in the food industry for product reformulation and portfolio evaluation [2,[13], [14], [15]]. Specifically, it is based on the concept of frailty prevention and consists of 4 recommended nutritional items (protein, dietary fiber, vitamin D, and calcium), 5 recommended food ingredients (fruits, vegetables, nuts, legumes, and dairy), and 3 limited nutritional items (energy, sugar, and sodium). Using MNPS-OA allows for a comprehensive evaluation of the nutritional value of a single food product based on the content of its 12 nutritional items. Importantly, NPSs should not only be developed but also validated from an epidemiological perspective by examining their association with health outcomes [16].

In Japan, applying conventional validation methodologies for NPSs is challenging [17]. This is primarily because large-scale cohort studies rarely capture detailed information on specific foods consumed by participants. Instead, dietary assessment tools such as the Food Frequency Questionnaire, the Dietary History Questionnaire (DHQ), and the Brief DHQ (BDHQ) are commonly used to provide estimates of daily intake at the diet level [[18], [19], [20], [21], [22]]. Applying food-based NPS rules directly to such data introduces several issues. Specifically, threshold-based scoring can truncate dose-response relationships, potentially distorting the evaluation of nutrient intake; aggregating energy penalties can lead to excessive deductions based on total energy intake, reducing the credibility of the score; and inconsistencies in food group classification can compromise reproducibility. Therefore, when validating food-based NPSs in Japan, it is essential to develop an approach that adapts the original NPS to diet-level data while maintaining conceptual validity and aligning with the context of Japan’s nutritional epidemiological studies [23].

In this study, we applied several modifications to the MNPS-OA to calculate diet quality scores using data from the Hatoyama Cohort Study and the Kusatsu Cohort Study: *1*) the original model, *2*) a model without energy limits (WEL), *3*) a model without nutrient caps (WC), and *4*) a model without both energy limits and nutrient caps (WEL-WC). The rationale for these modifications is described in Table 1. Construct validity was assessed through correlations among the modified models, and convergent validity was evaluated by comparing these scores with internationally recognized indices, Healthy Eating Index-2015 (HEI-2015) and Nutrient-Rich Food Index 9.3 (NRF9.3) [4,13,24,25]. We hypothesized that the modified MNPS-OA models would demonstrate improved construct and convergent validity. Ultimately, the choice of model for future applications will depend on study objectives and the type of data, requiring careful scientific evaluation and discussion.

## Methods

### Study population and nutritional assessments

This cross-sectional study was conducted using data from the Hatoyama Cohort Study and the Kusatsu Cohort Study. The study designs and protocols have been described in detail elsewhere [26,27]. In brief, the Hatoyama Cohort Study, initiated in 2010, enrolled 742 community-dwelling adults aged ≥65 y living in Hatoyama Town, Saitama Prefecture, Japan. Participants were selected through stratified random sampling based on age and residential area. The Kusatsu Cohort Study, initiated in 2002, targeted older adults in Kusatsu town, a rural area in northwestern Gunma Prefecture, Japan. The study population included National Health Insurance enrollees aged 65–74 y adults. Because common variables were collected in the Hatoyama Cohort Study in 2012 and the Kusatsu Cohort Study in 2013, these 2 datasets were combined to create pooled data. In total, 1130 adults participated. The nutritional intake of participants was assessed using the validated BDHQ, evaluating food intake, energy intake, and nutrient intake of the participants. Because the BDHQ cannot accurately capture sugar intake, we substituted the percentages of total sugars and added sugars from prior studies (total sugars: 10.7% energy for men, 13.5% energy for women; added sugars: 5.8% energy for men, 7.2% energy for women) [28]. This method allowed for a tentative estimation of sugar intake according to the participants’ energy intake [29].

As exclusion criteria, in addition to the 12 participants who did not attend the health checkup and did not provide written consent, 16 participants were excluded because their energy intake estimated from the BDHQ was suspected to be underestimated or overestimated (<600 kcal or >4000 kcal). Consequently, the final analysis of the population comprised 1102 participants [20]. Both studies were reviewed and approved by the Ethics Committee of the Tokyo Metropolitan Institute of Gerontology, and all participants provided written informed consent. The collaborative study was also reviewed and approved by the same committee (R25-017).

### HEI-2015 and NRF9.3 score calculation

The HEI-2015, developed by the USDA and the National Cancer Institute, assesses adherence to the 2015–2020 Dietary Guidelines for Americans (DGA) [24]. It includes 13 components: 9 adequacy components (e.g., fruits, vegetables, whole grains, dairy, protein foods, seafood and plant proteins, and fatty acids) and 4 moderation components (refined grains, sodium, added sugars, and saturated fats). Each component is scored per 1000 kcal, yielding a total score ranging from 0 to 100, with higher scores indicating closer compliance with the DGA. HEI-2015 is widely applied in nutritional epidemiology to evaluate overall diet quality and monitor population dietary trends [30]. In the HEI-2015 calculations, the intake of added sugars used values substituted from the energy ratio in prior research, and the intake of whole grains was assumed to be 0. Next, following previous research in Japan, American serving sizes were converted to Japanese volume equivalents, and the Japanese HEI-2015 score was calculated [31,32].

The NRF9.3 quantifies the nutrient density of foods and diets. It is based on 9 nutrients to encourage (protein, fiber, vitamins A, C, and D, calcium, iron, potassium, and magnesium) and 3 to limit (saturated fat, added sugars, and sodium). The score is calculated as the sum of the percentage daily values (%DV) of the beneficial nutrients minus the %DV of the limiting nutrients, standardized per 100 kcal or per reference energy intake. Unlike the food-based, guideline-oriented HEI-2015, NRF9.3 is nutrient-based and is particularly useful for comparing nutrient density across foods and dietary patterns. In previous studies, convergent validity of the food-based Meiji NPS was also evaluated against NRF9.3, ensuring methodological consistency; we therefore employed NRF9.3 in this study to allow direct comparability with earlier work and to reinforce the validation robustness. There are some missing values for certain nutrient intakes, and each was addressed using appropriate methods. Sugar intake was not based on individual-level data but estimated as an energy-based proportion derived from previous studies.

Both HEI-2015 and NRF9.3 have been validated for assessing diet quality in Japanese populations. Studies using BDHQ have demonstrated their reproducibility and validity, with higher scores consistently associated with favorable dietary patterns [32]. These findings support the use of these indices as robust measures for evaluating diet quality in Japanese epidemiological research.

### Introduction of the MNPS-OA and its modifications

The MNPS-OA was developed to address health issues specific to the Japanese older population and to reflect local nutrient intake patterns [2]. The MNPS-OA incorporates nutrients to limit (energy, sugars, and sodium), nutrients to encourage (protein, dietary fiber, calcium, and vitamin D), and food groups to encourage (fruits, vegetables, nuts, legumes, and dairy).

The nutrient caps are set for each component to promote appropriate intake. The MNPS-OA has shown moderate correlations with established indices of diet quality [NRF9.3: *r* = 0.60; Health Star Rating (HSR): *r* = 0.61], supporting its scientific validity and cultural relevance as a tool for assessing the nutritional value of foods in Japan.

In this study, the MNPS-OA was applied to assess overall diet quality. For all models, individual scores were calculated by summing points deducted for limiting nutrients and points added for encouraging nutrients and food groups. Higher scores indicate better overall dietary quality at the individual level. However, the MNPS-OA is not directly adaptable to diet-level data obtained from dietary questionnaires. Therefore, we created 4 models with interpretable and scientifically justified modifications: *1*) the original model, *2*) the WEL model, *3*) the WC model, and *4*) the WEL-WC model. Details of the scoring components are provided in Table 2, and Table 1 summarizes the specific modifications and the rationale for each change.

**TABLE 2:** Details for MNPS-OA

Nutritional factors		MNPS-OA Original model		MNPS-OA WEL model		MNPS-OA WC model		MNPS-OA WEL-WC model	
Factor types	Unit	RDVs	Cap (/RDVs, %)	RDVs	Cap (/RDVs, %)	RDVs	Cap (/RDVs, %)	RDVs	Cap (/RDVs, %)
Nutritional factors to limit	Energy (kcal)	2400	—	—	—	2400	—	—	—
	Sugar (g)	60	—	60	—	60	—	60	—
	Sodium (g)	7.5	—	7.5	—	7.5	—	7.5	—
Nutritional factors to encourage	Protein (g)	60	60 (100)	60	60 (100)	60 (100)	—	60 (100)	—
	Dietary fiber (g)	20	20 (100)	20	20 (100)	20 (100)	—	20 (100)	—
	Vitamin D (μg)	8.5	8.5 (100)	8.5	8.5 (100)	8.5 (100)	—	8.5 (100)	—
	Calcium (mg)	750	389.4 (52)	750	389.4 (52)	750 (100)	—	750 (100)	—
Food groups to encourage	Fruits (g)	200	113 (57)	200	113 (57)	200 (100)	—	200 (100)	—
	Vegetables (g)	350	84.7 (24)	350	84.7 (24)	350 (100)	—	350 (100)	—
	Nuts (g)	75	75 (100)	75	75 (100)	75 (100)	—	75 (100)	—
	Legumes (g)	100	57 (57)	100	57 (57)	100 (100)	—	100 (100)	—
	Dairy (g)	130	55 (42)	130	55 (42)	130 (100)	—	130 (100)	—

Abbreviations: MNPS-OA, Meiji NPS for Older Adults; NPS, Nutrient or Nutritional Profiling System; RDV, reference daily value.

**TABLE 1:** Modifications and reasons for the 4 models

#	Model	Modifications	Reasons
1	Original	—	—
2	WEL model	Excluding energy from the limit items	The intake of energy from diet is considered necessary for vital activities and is not an item that should be limited.
3	WC model	Excluding nutrient caps for recommended items and food ingredients (i.e., points are awarded up to the reference daily values)	Setting nutrient caps is a way to prevent overconsumption, but the diet should provide up to reference daily values of various nutritional items.
4	WEL-WC model	Excluding energy from the limit items and excluding nutrient caps for recommended items and food ingredients	The intake of energy from diet is considered necessary for vital activities and is not an item that should be limited. Setting nutrient caps is a way to prevent overconsumption, but the diet should provide up to reference daily values of various nutritional items.

Abbreviations: WC, without nutrient caps; WEL, without energy limit; WEL-WC, without energy limit and nutrient caps.

### Statistical analysis

Participant characteristics are presented as the median (interquartile range) for continuous variables (e.g., age) and as the number and percentage for categorical variables (e.g., sex). The *P* value indicates the difference between Hatoyama Cohort Study participants and Kusatsu Cohort Study participants for each variable. For continuous variables, the Mann–Whitney *U*-test was used; for categorical variables, the chi-square test or Fisher’s exact test was used.

This study uses Spearman’s rank correlation coefficient, considered the most methodologically appropriate, to validate multiple dietary quality scores (continuous variables). Specifically, Spearman’s correlation coefficients between the original model and 3 modified models were calculated to confirm the construct validity between models. Furthermore, Spearman’s correlation coefficients were calculated between the original model, the 3 modified models, HEI-2015, and NRF9.3 to confirm convergent validity. Additionally, Spearman’s rank correlation coefficient was used to examine correlations between each scale and nutrients. However, to address measurement errors in nutrients, each nutrient was processed using the density method [33]. Specifically, macronutrients were converted from daily intake to a percentage of energy intake by multiplying by the Atwater coefficients. For micronutrients, daily intake was converted to a unit of 1000 kcal/d [34]. Because the calculation of scores for each scale requires data on the subject’s daily intake, they were not adjusted.

All statistical analyses were performed using STATA version 16 (Stata Corp LLC, College Station). Two-tailed *P* values < 0.05 were considered statistically significant.

## Results

### Study population

This study included 1102 participants, 561 from the Hatoyama Cohort Study and 541 from the Kusatsu Cohort Study (Table 3). The median age was 73 y, and the sex distribution was approximately equal (50% men and 50% women). The median MNPS-OA scores were 124.5 in the original model, 205.3 in the WEL model, 302.6 in the WC model, and 391.6 in the WEL-WC model. The median HEI-2015 and NRF9.3 scores were 56.5 and 33.2, respectively.

**TABLE 3:** Characteristics of the population

	All participants (*n* = 1102)	Hatoyama study (*n* = 561)	Kusatsu study (*n* = 541)	*P* value
Age	73 (69, 78)	72.0 (69.0, 77.0)	73.0 (70.0, 78.0)	0.15
Sex				
Male	551 (50.0)	331 (59.0)	220 (40.7)	<0.001
Female	551 (50.0)	230 (41.0)	321 (59.3)	
BMI	23.1 (21.1, 25.0)	23.2 (21.2, 24.9)	23.0 (20.8, 25.2)	0.99
MMSE Score (*n* = 1064)	29 (28, 30)	29.0 (28.0, 30.0)	29.0 (27.0, 30.0)	0.76
Education year (*n* = 650)	9 (12, 15)	12.0 (11.0, 16.0)	10.0 (9.0, 12.0)	<0.001
Self-rated health				
Excellent	105 (9.5)	43 (7.7)	62 (11.5)	0.057
Good	820 (74.4)	436 (77.7)	384 (71.0)	
Fair	140 (12.7)	66 (11.8)	74 (13.7)	
Poor	37 (3.4)	16 (2.9)	21 (3.9)	
Chew ability				
Can chew and eat anything	666 (60.4)	361 (64.3)	305 (56.4)	0.013
Can chew and eat most foods	409 (37.1)	192 (34.2)	217 (40.1)	
Cannot chew many foods	26 (2.4)	8 (1.4)	18 (3.3)	
Can hardly chew any foods	1 (0.1)	0 (0.0)	1 (0.2)	
Alcohol consumption frequency				
Almost every day	260 (23.6)	164 (29.2)	96 (17.7)	<0.001
3–4 times/wk	93 (8.4)	51 (9.1)	42 (7.8)	
1–2 times/wk	69 (6.3)	41 (7.3)	28 (5.2)	
Rarely drink	239 (21.7)	106 (18.9)	133 (24.6)	
Formerly drank but stopped	68 (6.2)	32 (5.7)	36 (6.7)	
Never	373 (33.9)	167 (29.8)	206 (38.1)	
Smoking frequency				
Smokes	106 (9.6)	48 (8.6)	58 (10.7)	0.10
Smoked but stopped	376 (34.1)	207 (36.9)	169 (31.2)	
Never	620 (56.3)	306 (54.5)	314 (58.0)	
Number of medical conditions	2 (1, 3)	2.0 (1.0, 3.0)	1.0 (1.0, 3.0)	0.23
MNPS-OA (original model) score	124.5 (76.1, 168.1)	122.2 (73.3, 167.0)	126.7 (77.4, 169.8)	0.32
MNPS-OA (WEL model) score	205.3 (165.8, 242.0)	205.1 (165.6, 242.0)	205.4 (166.9, 242.0)	0.53
MNPS-OA (WC model) score	302.6 (239.9, 361.9)	303.7 (239.5, 364.9)	300.9 (241.4, 360.4)	0.80
MNPS-OA (WEL-WC model) score	391.6 (330.5, 442.3)	392.1 (330.8, 445.2)	390.4 (330.5, 439.1)	0.37
HEI-2015 score	56.5 (53.3, 59.2)	56.1 (53.0, 59.0)	57.1 (53.5, 59.4)	0.01
NRF9.3 score	33.2 (26.6, 40.1)	32.2 (26.3, 39.7)	34.1 (27.9, 40.3)	0.03

Participant characteristics are presented as the median (interquartile range) for continuous variables (e.g., age) and as the number and percentage for categorical variables (e.g., sex). The *P* value indicates the difference between Hatoyama Cohort Study participants and Kusatsu Cohort Study participants for each variable. For continuous variables, the Mann–Whitney *U*-test was used; for categorical variables, the chi-square test or Fisher’s exact test was used.

Abbreviations: HEI, Healthy Eating Index-2015; MMSE, Mini-Mental State Examination; MNPS-OA, Meiji NPS for Older Adults; NPS, Nutrient or Nutritional Profiling System; NRF9.3, Nutrient-Rich Food Index 9.3; WC, without nutrient caps; WEL, without energy limit; WEL-WC, without energy limit and nutrient caps.

### Correlation among MNPS-OA with modified models, and each model with HEI-2015 and NRF9.3

**FIGURE 1:**
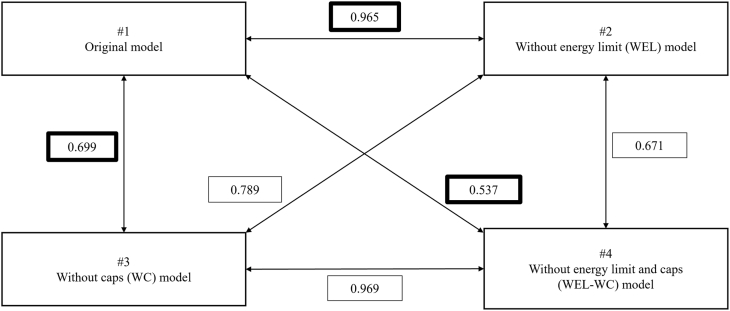
Construct validity of multiple Meiji NPS modifications models to the original model. NPS, Nutrient or Nutritional Profiling System.

**FIGURE 2:**
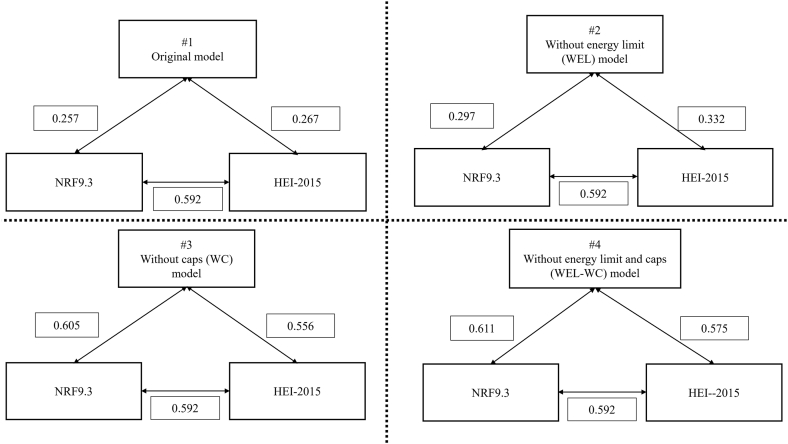
Convergent validity of multiple Meiji NPS modification models to HEI-2015 and NRF9.3, and correlation between HEI-2015 and NRF9.3. HEI, Healthy Eating Index-2015; NPS, Nutrient or Nutritional Profiling System; NRF9.3, Nutrient-Rich Food Index 9.3.

**FIGURE 3:**
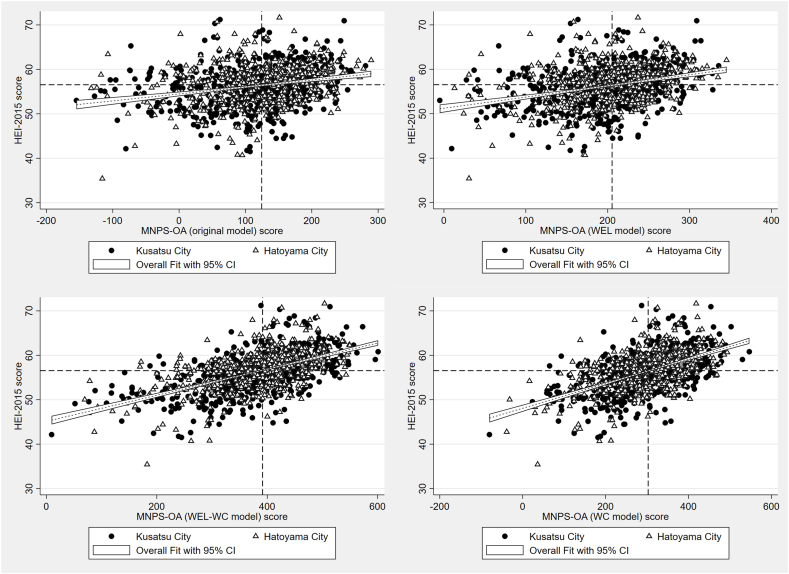
Visual examination of correlations between the Meiji NPS original model, WEL mode, WC model, and WEL-WC model for HEI-2015 score. HEI, Healthy Eating Index-2015; NPS, Nutrient or Nutritional Profiling System; WC, without nutrient caps; WEL, without energy limit; WEL-WC, without energy limit and nutrient caps.

**FIGURE 4:**
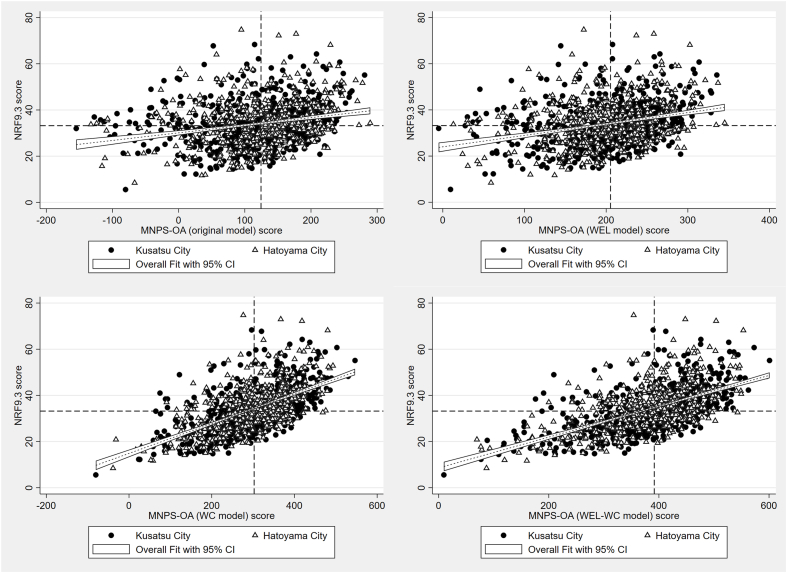
Visual examination of correlations between the Meiji NPS original model, WEL mode, WC model, and WEL-WC model for NRF9.3 score. NPS, Nutrient or Nutritional Profiling System; NRF9.3, Nutrient-Rich Food Index 9.3; WC, without nutrient caps; WEL, without energy limit; WEL-WC, without energy limit and nutrient caps.

Correlations were very high between the original and WEL models (*r* = 0.965), moderate for the WC model (*r* = 0.699), and lower for the WEL-WC model (*r* = 0.537). The correlation strengths with HEI-2015 were 0.267 for the original model, 0.332 for the WEL model, 0.556 for the WC model, and 0.575 for the WEL-WC model. The correlation strengths with NRF9.3 were 0.257 for the original model, 0.297 for the WEL model, 0.605 for the WC model, and 0.611 for the WEL-WC model. These results indicate that model specifications led to meaningful differences in convergence with established indices (Table 4, FIGURE 1, FIGURE 2). As shown in FIGURE 3, FIGURE 4, correlations with HEI-2015 and NRF9.3 were markedly higher when using the WC or WEL-WC models compared with the original model.

**TABLE 4:** Construct validity of multiple MNPS-OA models, and convergent validity to HEI-2015 and NRF9.3

Model variant	Construct validity	Convergent validity	
	Correlation with original model	Correlation with HEI-2015	Correlation with NRF9.3
Original	—	0.267	0.257
WEL model	0.965	0.332	0.297
WC model	0.699	0.556	0.605
WEL-WC model	0.537	0.575	0.611

Spearman’s rank correlation coefficients were calculated to assess the associations of the 3 modified models with the original model, as well as with HEI-2015 and NRF9.3.

Abbreviations: HEI, Healthy Eating Index-2015; MMSE, Mini-Mental State Examination; MNPS-OA, Meiji NPS for Older Adults; NPS, Nutrient or Nutritional Profiling System; NRF9.3, Nutrient-Rich Food Index 9.3; WC, without nutrient caps; WEL, without energy limit; WEL-WC, without energy limit and nutrient caps.

### Correlations between each model, HEI-2015, NRF9.3, and nutrients

As shown in Table 5, the coefficient ranges were –0.419 to 0.349 for the original model, –0.533 to 0.383 for the WEL model, –0.657 to 0.648 for the WC model, and –0.691 to 0.640 for the WEL-WC model. These findings indicate that the 4 models captured different ranges of association strength. In comparison, the HEI-2015 and NRF9.3 scores ranged from –0.418 to 0.679 and –0.605 to 0.932, respectively.

**TABLE 5:** Correlations between each model from MNPS-OA, HEI-2015, NRF9.3, and nutrients

Nutritional items	Unit	MNPS-OA sore				HEI-2015 score	NRF9.3 score
		Original model	WEL model	WC model	WEL-WC model		
Energy	kcal/d	−0.711	−0.519	−0.211	0.010	0.025	−0.020
Protein	g/1000 kcal/d	−0.008	−0.028	0.262	0.256	0.322	0.678
Fat	g/EN/d	−0.001	0.007	0.217	0.227	0.182	0.370
SFA	g/EN/d	0.015	0.029	0.205	0.216	−0.050	0.188
Carbohydrate	g/EN/d	0.152	0.139	−0.058	−0.084	−0.158	−0.259
Dietary fiber	g/1000 kcal/d	0.349	0.383	0.591	0.581	0.516	0.805
Vitamin A	μgRAE/1000 kcal/d	0.092	0.113	0.305	0.318	0.277	0.684
Vitamin D	μg/1000 kcal/d	−0.155	−0.177	0.068	0.074	0.231	0.458
Vitamin E	mg/1000 kcal/d	0.098	0.120	0.409	0.419	0.511	0.765
Vitamin K	μg/1000 kcal/d	0.304	0.349	0.515	0.516	0.384	0.670
Vitamin B1	mg/1000 kcal/d	0.218	0.238	0.548	0.548	0.479	0.835
Vitamin B2	mg/1000 kcal/d	0.288	0.282	0.508	0.474	0.369	0.764
Niacin	mgNE/1000 kcal/d	−0.025	−0.035	0.186	0.184	0.320	0.620
Vitamin B6	mg/1000 kcal/d	0.171	0.200	0.473	0.478	0.501	0.831
Vitamin B12	μg/1000 kcal/d	−0.190	−0.213	0.014	0.021	0.198	0.443
Folic acid	μg/1000 kcal/d	0.303	0.319	0.533	0.514	0.485	0.903
Vitamin C	mg/1000 kcal/d	0.279	0.309	0.566	0.564	0.605	0.838
Potassium	mg/1000 kcal/d	0.310	0.340	0.648	0.640	0.571	0.932
Calcium	mg/1000 kcal/d	0.186	0.188	0.526	0.512	0.398	0.707
Magnesium	mg/1000 kcal/d	0.231	0.244	0.553	0.542	0.514	0.876
Phosphorus	mg/1000 kcal/d	0.059	0.051	0.383	0.378	0.382	0.743
Iron	mg/1000 kcal/d	0.185	0.188	0.442	0.429	0.417	0.876
Zinc	mg/1000 kcal/d	0.094	0.077	0.306	0.293	0.271	0.655
Copper	mg/1000 kcal/d	0.297	0.307	0.462	0.442	0.405	0.721
Sodium	g/1000 kcal/d	−0.159	−0.269	−0.067	−0.121	0.159	0.335
Dietary Na/K ratio		−0.419	−0.533	−0.657	−0.691	−0.418	−0.605
Fruits	g/1000 kcal/d	0.243	0.293	0.493	0.506	0.679	0.422
Vegetables	g/1000 kcal/d	0.251	0.284	0.550	0.552	0.442	0.782
Legumes	g/1000 kcal/d	0.281	0.325	0.512	0.519	0.321	0.488
Dairy	g/1000 kcal/d	0.286	0.299	0.454	0.428	0.151	0.180

Spearman’s rank correlation coefficients were calculated to assess the associations of 4 models from MNPS-OA, HEI-2015, NRF9.3, and each nutrient.

Abbreviations: HEI, Healthy Eating Index-2015; MMSE, Mini-Mental State Examination; MNPS-OA, Meiji NPS for Older Adults; NPS, Nutrient or Nutritional Profiling System; NRF9.3, Nutrient-Rich Food Index 9.3; WC, without nutrient caps; WEL, without energy limit; WEL-WC, without energy limit and nutrient caps.

## Discussion

This study applied the MNPS-OA—the world’s first NPS developed specifically for older populations—to diet-level data. The findings suggest that the MNPS-OA can be used at the diet level with targeted and interpretable modifications. Removing nutrient caps on recommended nutrients markedly improved correlations with HEI-2015 and NRF9.3 (0.26–0.57), whereas removing energy limits had little effect (*r* ≈ 0.97 with the original MNPS-OA). Among the tested specifications, the uncapped model showed relatively stronger convergence with external indices, suggesting its potential suitability for diet-level applications.

An interesting finding of this study is that MNPS-OA demonstrated stronger correlations with the dietary Na/K ratio and dairy intake compared with HEI-2015 and NRF9.3. Dairy products are recognized as an important source of calcium in the Japanese diet, and incorporating dairy into traditional Japanese meals has been associated with reduced mortality risk [35]. The dietary Na/K ratio has recently gained global attention as a novel dietary factor, because it integrates both sodium and potassium intake and serves as a predictor for various health outcomes [[36], [37], [38]]. The stronger correlation of MNPS-OA with dairy intake likely reflects the inclusion of dairy as an explicit scoring component in the NPS. Similarly, the association with the dietary Na/K ratio may be attributable to the scoring approach that penalizes high salt intake while rewarding consumption of potassium-rich foods such as fruits, vegetables, and dairy. Although further investigation is warranted, MNPS-OA may better capture the actual dietary characteristics of Japanese older adults, suggesting its potential utility as a more culturally relevant diet quality index for this population.

In the MNPS-OA, nutrient caps were defined as the difference between the reference DVs and the median intakes of older adults from the 2019 National Health and Nutrition Survey in Japan [2,39]. Their purpose was to avoid rewarding excessive intake while encouraging correction of shortfalls. When nutrient caps were removed, points were awarded up to the full reference DVs, allowing diets richer in beneficial nutrients to score more fully and thereby improving correlations with HEI-2015 and NRF9.3. In contrast, removing energy limits had minimal impact. This can be explained structurally—because multiple components are integrated, dropping the penalty for one factor (energy) has limited influence—and conceptually, as energy is essential for life, is not scored in HEI-2015, and NRF9.3 is standardized per 100 kcal. Consequently, the very strong correlation between the original and WEL models is plausible, whereas the WC and WEL-WC models showed only moderate correlations with the original but improved convergence with HEI-2015 and NRF9.3. In addition, 3 sensitivity analyses were conducted to confirm the robustness of the findings. First, because the range of scores differed across diet quality indices, all scores were divided into deciles, and Spearman’s correlation coefficients were recalculated; the direction and strengths of correlations were almost identical to the main results. The correlation strengths with HEI-2015 were 0.553 for the WC model, and 0.570 for the WEL-WC model. The correlation strengths with NRF9.3 were 0.595 for the WC model, and 0.600 for the WEL-WC model. Second, correlations were examined separately by sex and by region to account for potential subgroup differences; again, the results were consistent with the main findings. The correlation strengths with HEI-2015 were 0.557 for the WC model, and 0.573 for the WEL-WC model, and correlation strengths with NRF9.3 were 0.581 for the WC model, and 0.607 for the WEL-WC model in male, respectively. The correlation strengths with HEI-2015 were 0.591 for the WC model, and 0.604 for the WEL-WC model, and correlation strengths with NRF9.3 were 0.534 for the WC model, and 0.561 for the WEL-WC model in male, respectively. The correlation strengths with HEI-2015 were 0.577 for the WC model, and 0.596 for the WEL-WC model, and correlation strengths with NRF9.3 were 0.621 for the WC model, and 0.643 for the WEL-WC model in Hatoyama city, respectively. The correlation strengths with HEI-2015 were 0.537 for the WC model, and 0.561 for the WEL-WC model, and correlation strengths with NRF9.3 were 0.585 for the WC model, and 0.577 for the WEL-WC model in Kusatsu town, respectively. Third, to further evaluate diet quality from a culturally relevant perspective, we used the Diet Quality Score for Japanese (DQSJ), which was specifically developed to assess dietary quality among Japanese individuals [40]. The DQSJ consists of 10 components: fruits, vegetables, whole grains, dairy, nuts, legumes, fish, red and processed meat, sugar-sweetened beverages, and sodium. Each component was scored from 0 to 3 according to sex-specific quartiles of intake, with higher scores indicating better diet quality. In this study, because intake data for refined grains and nuts were not available, these components were treated as nonconsumption and assigned 0 points when calculating the DQSJ. The total score ranged from 0 to 30. The correlation coefficients with the DQSJ score were 0.286 for the original model, 0.373 for the WEL model, 0.658 for the WC model, and 0.697 for the WEL-WC model. These results confirm our hypothesis: removing nutrient upper limits from the MNPS-OA prototype model strengthened correlations between the MNPS-OA modified model and both HEI-2015 and NRF9.3, while progressively enhancing positive correlations with nearly all nutrients and food groups. These results demonstrate the transformation of the food-based NPS into an NPS capable of evaluating diet-level data. Importantly, both the WC model and the WEL-WC model can assess diet quality at a moderate level with HEI-2015 and NRF9.3, demonstrating the convergent validity of the modified MNPS-OA. The concept of nutrient caps varies across profiling systems but consistently reflects context-specific calibration [[41], [42], [43]].

In the MNPS-OA, nutrient caps aimed to prevent over-crediting at the diet level, but their removal improved validity. In the HSR, for example, a “protein cap” limits protein points in products with high baseline negative points unless fruits or vegetables are present—an approach appropriate to Western contexts with generally high protein intake. Although these systems differ, both aim to prevent over-scoring beyond reasonable thresholds. Importantly, thresholds must be tailored to the target population [[44], [45], [46], [47]]. Because HSR was developed in Australia/New Zealand, applying it in Japan requires transparent adjustments aligned with local intakes. Our finding—that removing nutrient caps improved convergence—supports this principle. In MNPS-OA, nutrient caps were applied to evaluate the nutritional value of individual foods. However, findings from this study indicate that the WC model provides a more accurate assessment of diet quality at the diet level, as points accumulate progressively until multiple nutrient targets are achieved.

Improvements to MNPS-OA for diet-level data align with the food industry’s strategy of customizing models according to purpose, subject, and context [48,49]. Depending on the NPS concept, its components may differ, but the underlying philosophy remains similar: to scientifically evaluate the nutritional value of a single food, dish, or meal based on multiple nutrients, rather than the quantity of a single nutrient. MNPS-OA was initially designed for individual food assessment, but in this study, it was adapted for diet-level data within an epidemiological context [[50], [51], [52], [53], [54], [55], [56]]. Both approaches demonstrate the importance of profiling systems tailored to differences in target populations, cultures, and applications, contributing to the enhancement of nutritional assessment measures. Furthermore, applying NPS from food to meals is critical from the perspective of the food hierarchy [[57], [58], [59]]. This means evolving from the traditional nutrition science approach of evaluating individual nutrients to an era where NPS assesses foods, dishes, and meals [60,61].

The next step in MNPS-OA validation is to examine criterion and predictive validity—whether higher MNPS-OA scores are associated with geriatric or age-related syndromes in older adult population [7,[62], [63], [64], [65]]. Given the extensive literature linking diet indices to these outcomes in Japan and elsewhere, this represents a feasible and important direction [[66], [67], [68], [69], [70]]. Importantly, recognizing the characteristics of existing NPSs—including target regions, populations, and thresholds—and adapting them to local dietary cultures and intake patterns enhances validity and ensures scientific applicability for implementation [71]. To our knowledge, MNPS-OA is the only NPS specifically designed for older adults; therefore, future research may explore tailoring MNPS-OA for multiple aging societies worldwide to ensure contextual relevance [72]. Furthermore, no studies worldwide have applied food-based NPSs to diet-level data derived from dietary questionnaires. This study was designed within the context of Japanese nutritional epidemiology study and aims to provide a novel methodological contribution, serving as a bridge between NPS science and epidemiological practice, and advancing NPS research in Japan [13,16,73].

### Strengths and limitations

The primary strength of this study lies in being the first to systematically evaluate the construct and convergent validity of a NPS specifically designed for older adults when applied at the diet level. Our findings demonstrate that an NPS originally developed for assessing individual foods can be effectively adapted to diet-level data derived from dietary questionnaires—the primary tools used in large-scale epidemiological studies—thereby enhancing reproducibility and practical applicability. Furthermore, by comparing algorithmic specifications, this study emphasizes scientific interpretability and employs a hypothesis-driven validation approach.

Several limitations should be acknowledged. Dietary data were obtained from the BDHQ, and nutrient amounts were treated as true intake values when constructing each scale; therefore, some underestimation or overestimation cannot be ruled out. Although BDHQ has limitations in estimating absolute intake, it is a validated tool widely used in Japan. To minimize bias, nutrient data were processed using density methods when examining correlations with the scale. Our primary aim was to develop a scale for relative evaluation rather than to ensure absolute precision of individual scores, for which BDHQ data are considered appropriate. Second, although HEI-2015 and NRF9.3 are widely used internationally, they may not fully capture the unique characteristics of the Japanese diet. To address this, we also examined correlations with the DQSJ, which showed consistent and favorable results. Third, there are some missing values for certain nutrient intakes, and each was addressed using appropriate methods. Sugar intake was not based on individual-level data but estimated as an energy-based proportion derived from previous studies. In MNPS-OA, nut intake was assumed to be 0, and in HEI-2015, whole grain intake was assumed to be 0. Japan does not have a comprehensive database for sugar content. Furthermore, the National Health and Nutrition Survey reports that the intake of nuts and whole grains is either 0 or extremely low.

Future research should confirm the criterion and predictive validity of MNPS-OA, evaluate its performance in diverse populations and dietary assessment methods, and explore its potential for integration into public health strategies and clinical practice.

In conclusion, this study demonstrates that MNPS-OA, with targeted modifications, can be applied to diet-level data, bridging the gap between nutrient profiling science and epidemiological practice. These findings support its potential use in public health surveillance and product reformulation strategies for aging societies.

## Author contributions

The authors’ responsibilities were as follows – TY, YK, YY, HR: study design; TY, YK, HR: provision of data sets; TY, WR: statistical analysis of data; TY: preparation of tables and first draft of the manuscript; YK, WR, HR: critical review; all authors: commented on drafts, read and approved the final manuscript.

## Data availability

The data described in the manuscript, code book, and analytic tool will be made available upon request and with permission from the Research Team for Social Participation and Healthy Aging at the Tokyo Metropolitan Institute for Geriatrics and Gerontology.

## Funding

The Hatoyama Cohort Study in 2012 and the Kusatsu Cohort Study in 2013 were implemented under the support of a Grant-in-Aid for Scientific Research ([B] number 24390173) from the Japan Society for the Promotion of Science, research grants from the Research Institute of Science and Technology for Society (RISTEX), the Japan Science and Technology Agency, and the towns of Hatoyama and Kusatsu. This joint research project is funded by Meiji Co., Ltd.

## Conflict of interest

TY and RW are full-time employees of Meiji Co. Ltd. The other authors report no conflicts of interest.
